# Association between neutrophil percentage-to-albumin ratio (NPAR), neutrophil-to-lymphocyte ratio (NLR), platelet-to-lymphocyte ratio (PLR) and long-term mortality in community-dwelling adults with heart failure: evidence from US NHANES 2005–2016

**DOI:** 10.1186/s12872-023-03316-6

**Published:** 2023-06-21

**Authors:** Chia-Chen Wu, Chia-Hui Wu, Chien-Ho Lee, Cheng-I Cheng

**Affiliations:** 1 Division of Thoracic and Cardiovascular Surgery, Department of Surgery, Kaohsiung Chang Gung Memorial Hospital, Chang Gung University College of Medicine, Kaohsiung City, Taiwan; 2 Department of Medical Imaging, Kaohsiung Medical University Hospital, Kaohsiung Medical University, Kaohsiung, 807 Taiwan; 3 Division of Cardiology, Department of Internal Medicine, Kaohsiung Chang Gung Memorial Hospital, Chang Gung University College of Medicine, 123 Tapei Rd., Niaosung District, Kaohsiung City, 833 Taiwan

**Keywords:** Neutrophil percentage-to-albumin ratio (NPAR), Neutrophil-to-lymphocyte ratio (NLR), Platelet-to-lymphocyte (PLR), Heart failure (HF), Mortality, National Health and Nutrition Examination Survey (NHANES)

## Abstract

**Background:**

Heart failure (HF) continues to be the major cause of hospitalizations. Despite numerous significant therapeutic progress, the mortality rate of HF is still high. This longitudianl cohort study aimed to investigate the associations between hematologic inflammatory indices neutrophil percentage-to-albumin ratio (NPAR), neutrophil-to-lymphocyte ratio (NLR), platelet-to-lymphocyte ratio (PLR), and all-cause mortality in community-dwelling adults with HF.

**Methods:**

Adults aged 20 and older with HF in the US National Health and Nutrition Examination Survey (NHANES) database 2005–2016 were included and were followed through the end of 2019. Univariate and multivariable Cox regression analyses were performed to determine the associations between the three biomarkers and all-cause mortality. The receiver operating characteristics (ROC) curve analysis was conducted to evaluate their predictive performance on mortality.

**Results:**

A total of 1,207 subjects with HF were included, representing a population of 4,606,246 adults in the US. The median follow-up duration was 66.0 months. After adjustment, the highest quartile of NPAR (aHR = 1.81, 95%CI: 1.35, 2.43) and NLR (aHR = 1.59, 95%CI: 1.18, 2.15) were significantly associated with increased mortality risk compared to the lowest quartile during a median follow-up duration of 66.0 months. Elevated PLR was not associated with mortality risk. The area under the ROC curve (AUC) of NPAR, NLR, and PLR in predicting deaths were 0.61 (95%CI: 0.58, 0.65), 0.64 (95%CI: 0.6, 0.67), and 0.58 (95%CI:0.55, 0.61), respectively.

**Conclusions:**

In conclusion, elevated NPAR and NLR but not PLR are independently associated with increased all-cause mortality among community-dwelling individuals with HF. However, the predictive performance of NPAR and NLR alone on mortality was low.

## Background

More than 6 million persons in the United States (US) suffer from the clinical condition of heart failure (HF), and its prevalence is expected to rise to greater than 8 million by 2030 [1, 2]. Importantly, HF continues to be the primary cause of hospitalizations in older persons, and mortality rates are still high despite significant progress in understanding its etiology and related targeted therapy approaches [3]. Specifically, the 5-year survival rate for patients with newly diagnosed HF was less than 50% in a recent large cohort study, and the 5-year survival rate among patients admitted to the hospital at the time of diagnosis was less than 40% [4].

Biomarkers play a significant role in the prognostic evaluation of patients with HF. In some recent investigations, N-terminal pro BNP (NT-proBNP) and troponin surpassed other developing biomarkers in the prediction of unfavorable outcomes of acute HF [5, 6]. It is indicated that serial measures taken throughout the hospital stay with multi-marker models may yield better risk prediction [7].

Among the developing biomarkers, complete blood inflammation markers, mainly the neutrophil-to-lymphocyte ratio (NLR) and the platelet-to-lymphocyte-ratio (PLR), are regarded as quick and critical indicators in the assessment of the presence of subclinical inflammation in many diseases [8–11]. For HF, several studies have demonstrated the prognostic significance of NLR and PLR for in-hospital and long-term mortality in patients with HF [10, 11].

Recently, another blood marker derived from the routine blood sample, the neutrophil percentage-to-albumin ratio (NPAR), combines blood neutrophils and albumin and has been demonstrated to be a potentially useful prognostic indicator for mortality in patients with acute myocardial infarction (AMI) [12], cardiogenic shock [13], coronary artery disease [14], as well as HF in the intensive care unit [15].

However, to our knowledge, no previous studies have yet explored the prognostic value of NPAR in community-dwelling individuals with HF. Therefore, this study aimed to investigate the association between NPAR and long-term all-cause mortality in HF patients living in the community. Using a large, nationally representative data set, we also sought to evaluate the predictive significance of NPAR on HF mortality and compare it to the other two well-known hematologic markers, NLR and PLR..

## Materials and methods

### Data source

The secondary data from the United States National Health and Nutrition Examination Survey (NHANES) database were evaluated in this cross-sectional investigation. The National Center for Health Statistics (NCHS), a division of the Centers for Disease Control and Prevention (CDC) in the US, is the source of the NHANES program (http://www.cdc.gov/nchs/nhanes). This ongoing survey series combines interviews and examinations to assess adults’ and children’s health and nutritional status. The NHANES employs a complex, multistage design to collect and analyze data representative of the US’s non-institutionalized national population. NHANES participants complete a household interview before being invited to a comprehensive examination in a mobile examination center (MEC), which includes a physical exam, specialized measurements, and laboratory tests. Therefore, employing NHANES data enables trustworthy, multidimensional, and population-level studies on health-related topics.

### Ethics considerations

The NHANES program was examined and approved by the NCHS Research Ethics Review Board, and each survey respondent signed an informed consent form. The NCHS permits researchers to utilize the data made available for research purposes. All NHANES data made public by the NCHS are also de-identified, and the data are kept anonymous during analysis. Therefore, the secondary data analysis described in this study did not require any additional ethical approval or informed permission. The NCHS Research Ethics Review Board Approval details can be found on the NHANES website (https://www.cdc.gov/nchs/nhanes/irba98.htm).

### Study population

Participants’ data were extracted from eight released cycles of the NHANES from 2003 to 2018. The inclusion criteria were individuals aged ≥ 20 years with HF, defined by individuals’ report of having been diagnosed with congestive HF by a doctor in the questionnaire on health conditions (details of the questionnaire section are available on: https://wwwn.cdc.gov/Nchs/Nhanes/2013-2014/MCQ_H.htm). This approach to identifying HF was utilized in various prior NHANES studies [16, 17]. Subjects without complete data on the main study variables or mortality status during follow-up were excluded from the study cohort.

### Study variables

#### Assessment of study endpoint

In this study, we observed all-cause mortality, defined as death from any cause, as the study endpoint. Participants’ mortality status was validated by linking to the National Death Index (NDI) up to the end of 2019 (https://www.cdc.gov/nchs/data-linkage/mortality-public.htm).

#### NLR, PLR, and NPAR

The NHANES CBC Profile was used to acquire the hematologic parameters. The methods used to derive CBC parameters are based on the Beckman Coulter method of counting and sizing, in combination with an automatic diluting and mixing device for sample processing and a single beam photometer for hemoglobinometry. The WBC differential uses VCS technology.

The albumin level was determined using the NHANES Standard Biochemistry Profile. The LX20 uses a bichromatic digital endpoint approach to quantify albumin concentration. In the reaction, the albumin combines with Bromocresol Purple (BCP) reagent to form a complex. The system monitors the change in absorbance at 600 nm. The amount of albumin in the sample is directly proportional to the change in absorbance.

By dividing the total absolute neutrophil counts by the total absolute lymphocyte counts, the NLR was determined for each participant. The neutrophil percentage was determined as the proportion of neutrophils in white blood cells, and PLR was computed by dividing the total platelet counts by the total absolute lymphocyte counts [10]. The NPAR was calculated as the neutrophil percentage as the numerator divided by albumin using the same blood samples drawn according to the formula: (Neutrophil percentage (%) * 100/Albumin (g/dl)) [12]. Details descriptions of the laboratory methodology are available at: https://wwwn.cdc.gov/Nchs/Nhanes/2003-2004/L25_C.htm.

To determine the relationship between the indices and mortality, we treated the indices as continuous variables and in quartiles in the analyses.

#### Covariates

Using the Family and Sample Person Demographics questionnaires and the Computer-Assisted Personal Interviewing (CAPI) system (Confirmit Corp. New York, USA), in-person interviews were conducted by trained interviewers to gather data on age, gender, race, income-to-poverty ratio, marital status, and level of education (https://wwwn.cdc.gov/Nchs/Nhanes/2015-2016/DEMO_i.htm).

Body mass index (BMI) was determined by dividing a person’s weight in kilograms by their height in meters squared from NHANES examination measurements, and was classified into four categories: <18.5, 18.5 to 24.9, 25 to 29.9, and ≥ 30 kg/m^2^. Standing height was measured with a fixed stadiometer, and body weight was determined with an electronic load cell scale.

Participants’ smoking status was divided into three categories: current smoker, former smoker, and non-smoker. Specifically, a person with lifetime smoking of fewer than 100 cigarettes was a non-smoker; with lifetime smoking > 100 cigarettes but not currently a smoker was a former smoker; and with lifetime smoking > 100 cigarettes and responding “yes” to the question: “Do you smoke now?” was a current smoker.

Participants’ responses to the survey question “How old were you when you were first told you had congestive heart failure?” and their age at the survey time were used to compute the number of years since the HF diagnosis (https://wwwn.cdc.gov/Nchs/Nhanes/2017-2018/MCQ_J.htm).

The following questions were used to identify participants who had diabetes [18], and they were excluded from the study cohort: “Are you taking insulin?” “Did a doctor tell you that you have diabetes?” “Do you take pills to lower blood sugar?” (https://wwwn.cdc.gov/Nchs/Nhanes/2017-2018/DIQ_J.htm) or having an HbA1c ≥ 6.5%, a fasting glucose ≥ 126 mg/dL, or a glucose level ≥ 200 mg/dL in oral glucose tolerance test (OGTT) as recorded in the laboratory data file of the NHANES (https://wwwn.cdc.gov/Nchs/Nhanes/2017-2018/GLU_J.htm).

Blood pressure was measured three times using a standardized methodology, and the average was used for all analyses. Hypertension was defined as answering “yes” to the following questions: “Were you told on two or more different visits that you had hypertension, also called high blood pressure?” or “Because of your (high blood pressure/hypertension), have you ever been told to take prescribed medicine?” (https://wwwn.cdc.gov/Nchs/Nhanes/2017-2018/BPQ_J.htm), or with an average of three consecutive measures on systolic blood pressure (SBP) ≥ 140 mmHg, or with an average of three successive measurements on diastolic blood pressure (DBP) ≥ 90 mmHg (https://wwwn.cdc.gov/Nchs/Nhanes/2017-2018/BPX_J.htm).

The following question characterized coronary heart disease, history MI and stroke: “Has a doctor or other health professional ever told you that you have (disease)?” (https://wwwn.cdc.gov/Nchs/Nhanes/2017-2018/MCQ_J.htm).

Similarly, chronic obstructive pulmonary disease (COPD) was identified by people reporting chronic bronchitis or emphysema to a doctor or other health practitioner (https://wwwn.cdc.gov/Nchs/Nhanes/2017-2018/MCQ_J.htm).

The 4-variable Modification of Diet in Renal Disease (MDRD) Study equation was used to calculate the glomerular filtration rate (GFR) from re-calibrated serum creatinine. Here, we applied the standardized creatinine-based IDMS-traceable MDRD Study equation: GFR = 175 × (standardized serum creatinine)-1.154 × (age)-0.203 × 0.742 (for female) × 1.212 (for African American). Estimated GFR is reported in ml/min/1.73m^2^. An eGFR of less than 60 ml/min/1.73 m^2^ was used to identify chronic kidney disease (CKD) [19].

Participants with dyslipidemia were defined by at least one of the following questions: “To lower your blood cholesterol, have you ever been told by a doctor or other health professional... to take prescribed medicine?” “Have you ever been told by a doctor or other health professional that your blood cholesterol level was high?” (https://wwwn.cdc.gov/Nchs/Nhanes/2017-2018/BPQ_J.htm) or a serum total cholesterol level ≥ 200 mg/dL, HDL-c < 40 mg/dl for men, HDL-c < 50 mg/dl for women, LDL-c ≥ 130 mg/dl, or triglyceride ≥ 150 mg/dl [20].

Trouble sleeping was identified through the question: “Have you ever been told by a doctor or other health professional that you have trouble sleeping?”

Data on the use of HF medications include beta-blockers, angiotensin-converting enzyme inhibitors (ACEI) or angiotensin receptor blockers (ARB), aldosterone antagonists, vasodilators, and diuretics. During the household interview, survey participants are asked if they have taken a medication that required a prescription in the past 30 days. Those who respond “yes” must show the interviewer the medication containers for all products used. Doses of medication were not collected. Details of the data collection can be found at: https://wwwn.cdc.gov/Nchs/Nhanes/2003-2004/RXQ_RX_C.htm.

Hemoglobin, HbA1c, C-reactive protein (CRP), total cholesterol, high-density lipoprotein-cholesterol (HDL-C), low-density lipoprotein-cholesterol (LDL-C), and triglycerides were measured using blood collected during the study visit (https://wwwn.cdc.gov/Nchs/Nhanes/2017-2018/BIOPRO_J.htm).

### Statistical analysis

Specific sample weights (WTSAF2YR), stratum (SDMVSTRA), and cluster (SDMVPSU) were used to assure national representation. Continuous data are presented as mean ± standard deviations (SD) for demographic data and median (interquartile range, IQR) for medical history and laboratory data; categorical variables are presented as n (%). Differences between patients who died and those who did not die throughout the study follow-up period were compared using the Wilcoxon rank sum test for continuous data and Pearson’s Chi-squared test for categorical data. Univariate and multivariable Cox regression analyses were performed to determine the associations between all-cause mortality and the biomarkers. Patients were followed until the end of 2019 to determine mortality status. Results are presented as hazard ratio (HR) and corresponding 95% confidence interval (CI). A receiver operating characteristics (ROC) curve analysis was conducted to evaluate the performances of the three markers (i.e., NPAR, NLR, and PLR) in predicting all-cause mortality. Results are shown in the area under the ROC curve (AUC) with corresponding 95% CI with sensitivity and specificity. All P values are two-sided and considered significant when P < 0.05. All data arrangements and analyses were performed using R studio software [21].

## Results

### Study cohort selection

**Fig. 1:**
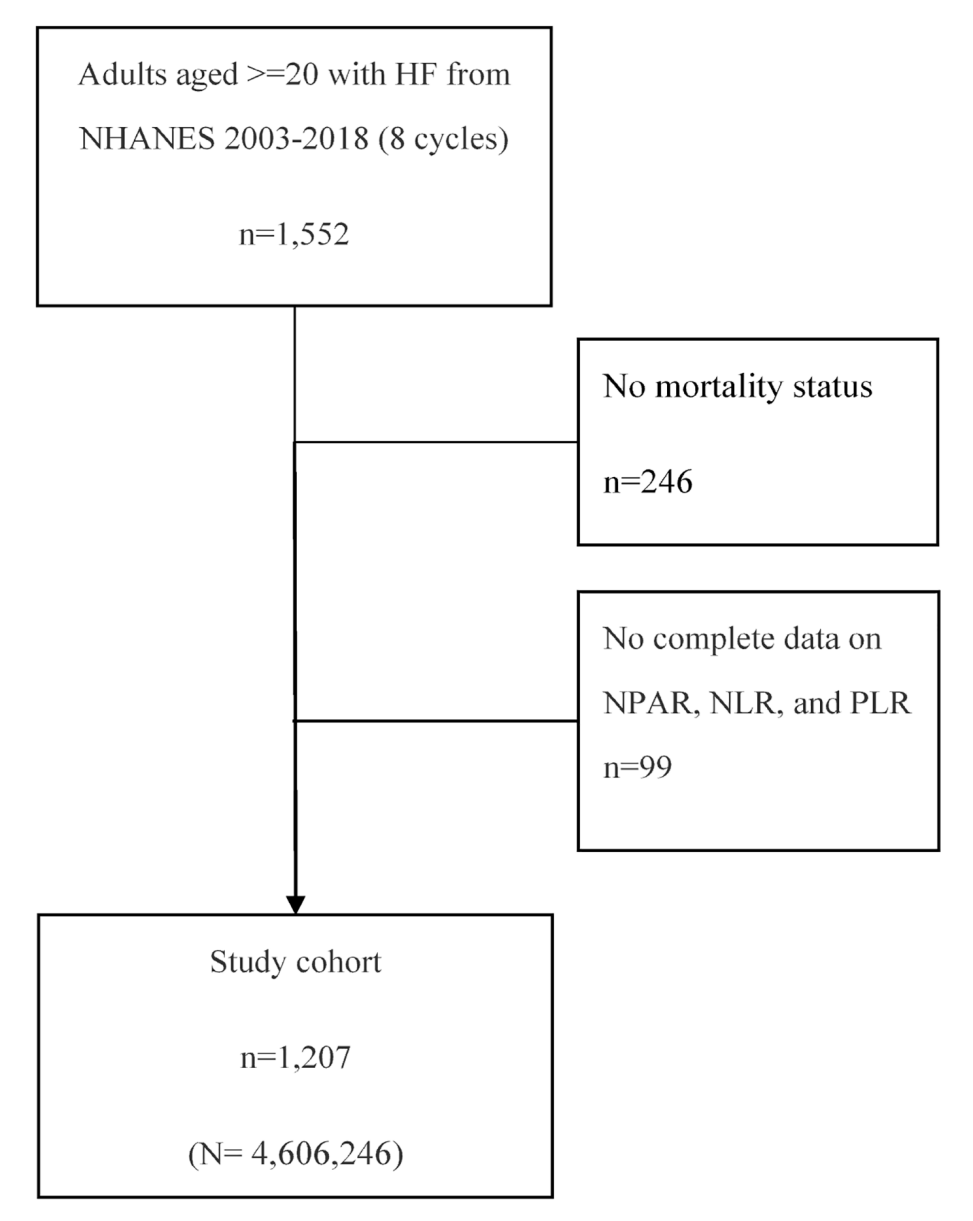
Flow diagram of study cohort selection

A total of 1,552 community-dwelling individuals aged 20 years and older with HF were identified in the NHANES database. After excluding patients without complete information on main variables and mortality status at follow-up, 1,207 subjects were included as the primary cohort. This sample represents a population size of 4,606,246 community-dwelling adults in the US. The flow diagram of the study inclusion and exclusion process is presented in Fig. 1.

### Characteristics of the study cohort

The characteristics of the study cohort are summarized in Table 1. During a median follow-up time of 66 months, there were 540 deaths. Subjects who deceased were older (72.2 ± 9.9 vs. 63.3 ± 13.0, p < 0.001). More males (60% vs. 54%, p = 0.038), non-Hispanic Whites (68% vs. 44%, p < 0.001), and subjects with lower BMI (30.5 ± 7.6 vs. 32.9 ± 8.4, p < 0.001) have not survived. Concerning medical comorbidities, greater proportions of the individuals with a history of stroke (23% vs. 18%, p = 0.029), COPD 22% vs. 16%, p = 0.003), and CKD (58% vs. 33%, < 0.001) died during the follow-up period (Table 1).

**Table 1:** Characteristics of the study cohort

Study variables	Total (N = 1,207)	Alive (N = 667)	Deceased (N = 540)	*P* value ^b^
Follow-up duration, months	66.0 (35.0,105.5)	77.0 (40.5,124.5)	56.0 (30.0,89.0)	
Demography				
Age, years	67.3 ± 12.5	63.3 ± 13.0	72.2 ± 9.9	**< 0.001**
20–39	42 (3.5%)	38 (5.7%)	4 (0.7%)	
40–49	90 (7.5%)	74 (11%)	16 (3.0%)	
50–59	143 (12%)	105 (16%)	38 (7.0%)	
60–69	321 (27%)	199 (30%)	122 (23%)	
70–79	334 (28%)	171 (26%)	163 (30%)	
80+	277 (23%)	80 (12%)	197 (36%)	
Males	689 (57%)	363 (54%)	326 (60%)	**0.038**
Race				**< 0.001**
Non-Hispanic White	664 (55%)	295 (44%)	369 (68%)	
Non-Hispanic Black	288 (24%)	191 (29%)	97 (18%)	
OT-Hispanic White	84 (7.0%)	68 (10%)	16 (3.0%)	
Other/unknown	171 (14%)	113 (17%)	58 (11%)	
BMI, kg/m^2^	31.8 ± 8.1	32.9 ± 8.4	30.5 ± 7.6	**< 0.001**
<18.5	15 (1.2%)	6 (0.9%)	9 (1.7%)	
18.5–24.9	212 (18%)	96 (14%)	116 (21%)	
25.0-29.9	342 (28%)	172 (26%)	170 (31%)	
≥30	638 (53%)	393 (59%)	245 (45%)	
Poverty income ratio > 1 ^a^	834 (75%)	446 (73%)	388 (77%)	0.106
Married or living with a partner ^a^	620 (51%)	358 (54%)	262 (49%)	0.075
Attended high school or above ^a^	764 (63%)	451 (68%)	313 (58%)	**< 0.001**
Smoking status ^a^				**0.01**
Never	468 (39%)	268 (40%)	200 (37%)	
Former	509 (42%)	257 (39%)	252 (47%)	
Current	229 (19%)	141 (21%)	88 (16%)	
Medical history				
Years since first diagnosed with HF, years ^a^	7.0 (2.0, 14.0)	7.0 (3.0, 14.0)	6.5 (2.0, 14.0)	0.335
≥ 5 years ^a^	749 (63%)	421 (64%)	328 (62%)	0.559
DM	556 (46%)	299 (45%)	257 (48%)	0.338
Hypertension ^a^	999 (84%)	561 (85%)	438 (82%)	0.21
Coronary heart disease	565 (47%)	306 (46%)	259 (48%)	0.47
History of MI	532 (44%)	281 (42%)	251 (46%)	0.13
History of stroke	239 (20%)	117 (18%)	122 (23%)	**0.029**
COPD	225 (19%)	104 (16%)	121 (22%)	**0.003**
CKD	537 (44%)	223 (33%)	314 (58%)	**< 0.001**
SBP, mmHg ^a^	129.3 (116.0, 144.7)	129.3 (116.0, 143.3)	130.0 (116.0, 146.7)	0.521
DBP, mmHg ^a^	66.0 (58.0, 76.0)	68.0 (60.0, 77.3)	64.0 (55.2, 73.3)	**< 0.001**
GFR, mL/min/1.73m^2^	63.4 (48.2, 80.6)	70.2 (55.1, 87.0)	55.9 (41.4, 71.0)	**< 0.001**
Trouble sleeping ^c^	518 (49%)	330 (52%)	188 (44%)	**0.009**
Dyslipidemia	1035 (86%)	578 (87%)	457 (85%)	0.316
Medication				
ACEI/ARB	706 (58%)	390 (58%)	316 (59%)	0.987
Aldosterone antagonist	99 (8.2%)	50 (7.5%)	49 (9.1%)	0.321
Beta Blocker	727 (60%)	394 (59%)	333 (62%)	0.359
Diuretics	595 (49%)	265 (40%)	330 (61%)	**< 0.001**
Vasodilators	123 (10%)	63 (9.4%)	60 (11%)	0.341
Laboratory measures				
NPAR	15.0 (13.2,17.0)	14.6 (12.7,16.3)	15.5 (14.0,17.7)	**< 0.001**
Q1 (< 13.2)	302 (25%)	207 (31%)	95 (18%)	**< 0.001**
Q2 (13.2 to < 15.0)	297 (25%)	166 (25%)	131 (24%)	
Q3 (15.0 to < 17.0)	306 (25%)	170 (25%)	136 (25%)	
Q4 (≥ 17.0)	302 (25%)	124 (19%)	178 (33%)	
PLR	116.7 (89.5,156.9)	111.3 (86.5,144.7)	124.6 (95.4,170.7)	**< 0.001**
Q1 (< 89.5)	302 (25%)	186 (28%)	116 (21%)	**< 0.001**
Q2 (89.5 to < 116.7)	301 (25%)	182 (27%)	119 (22%)	
Q3 (116.7 to < 156.9)	302 (25%)	168 (25%)	134 (25%)	
Q4 (≥ 156.9)	302 (25%)	131 (20%)	171 (32%)	
NLR	2.4 (1.7,3.3)	2.1 (1.5,2.9)	2.7 (1.9,3.8)	**< 0.001**
Q1 (< 1.7)	302 (25%)	213 (32%)	89 (16%)	**< 0.001**
Q2 (1.7 to < 2.4)	301 (25%)	178 (27%)	123 (23%)	
Q3 (2.4 to < 3.3)	297 (25%)	151 (23%)	146 (27%)	
Q4 (≥ 3.3)	307 (25%)	125 (19%)	182 (34%)	
Platelet, 10^3^/uL	213.0 (178.0, 263.5)	216.0 (178.0, 263.0)	211.0 (178.0, 265.0)	0.318
Lymphocyte, 10^3^/uL	1.8 (1.4, 2.4)	2.0 (1.5, 2.5)	1.7 (1.3, 2.3)	**< 0.001**
Neutrophil, 10^3^/uL	4.4 (3.4, 5.6)	4.2 (3.3, 5.4)	4.7 (3.6, 5.9)	**< 0.001**
Neutrophil percentage, %	61.3 (54.3, 67.4)	59.4 (53.0, 65.6)	63.7 (57.0, 69.6)	**< 0.001**
WBC, 10^3^/uL	7.3 (6.0, 8.8)	7.2 (5.9, 8.6)	7.4 (6.1, 8.9)	**0.026**
Albumin, g/dL	4.1 (3.8, 4.3)	4.1 (3.9, 4.3)	4.1 (3.8, 4.3)	0.057
Hemoglobin, g/dL	13.6 (12.5, 14.7)	13.8 (12.7, 14.7)	13.5 (12.4, 14.7)	**0.021**
CRP, mg/dL^a^	0.7 (0.2, 2.3)	1.2 (0.3, 3.6)	0.4 (0.2, 1.1)	**< 0.001**
Total Cholesterol, mg/dL	170.0 (143.0, 204.0)	171.0 (143.0, 205.5)	169.0 (142.0, 203.0)	0.665
HDL-c, mg/dL	46.0 (38.0, 57.0)	45.0 (38.0, 56.0)	47.0 (39.0, 58.0)	0.651
LDL-c, mg/dL ^a^	93.0 (70.0, 121.0)	95.5 (71.2, 122.8)	89.0 (68.0, 116.0)	0.115
TG, md/dL ^a^	122.0 (86.0, 179.0)	124.0 (85.5, 173.0)	121.5 (86.0, 183.8)	0.644
HbA1c, %	5.9 (5.5, 6.7)	5.9 (5.6, 6.6)	5.9 (5.5, 6.7)	0.714

Data are presented as mean ± SD, n (%), or median with interquartile range (IQR).

Abbreviation:

ACEI, angiotensin-converting enzyme inhibitors; ARB, angiotensin receptor blockers; SD, standard deviation; BMI, body mass index; HF, heart failure; COPD, chronic obstructive pulmonary disease; SBP, systolic blood pressure; DBP, diastolic blood pressure; MI, myocardial infarction; CKD, chronic kidney disease; NPAR, Neutrophil-percentage-to-albumin ratio; PLR, platelet-to-lymphocyte ratio; NLR, neutrophil-to-lymphocyte ratio; CRP, C-reactive protein; HDL-c, high-density lipoprotein cholesterol; LDL, low-density lipoprotein cholesterol; TG, triglycerides; Q, quartile

^a^ Missing data: poverty income ratio (8%), school attendance (0.1%), smoking status (0.1%), years since first diagnosis with HF (0.01%), hypertension (0.1%), blood pressure (5.0%), CRP (25%), total cholesterol (0.1%), LDL (52%), TG (51%), and HbA1c (0.2%)

^b^ P value was derived using the Wilcoxon rank sum test for continuous data and Pearson’s Chi-squared test for categorical data. Bold P values indicate significance

^c^ Not available in 2003–2004 cycle

### Associations between NPAR, NLR, PLR, and all-cause mortality in individuals with HF

Associations between NPAR, NLR, PLR, and all-cause mortality in patients with HF are summarized in Table 2. When assessed as continuous variables, after adjusting for age, gender, race, BMI, and comorbid conditions including DM, CKD, COPD, history of MI, and diuretics usage in the multivariable model, per unit increase of NPAR (aHR = 1.08, 95%CI: 1.05–1.12) and NLR (aHR = 1.05, 95%CI: 1.02–1.08) were significantly associated with greater all-cause mortality risk, whereas PLR was not. In addition, compared with the lowest quartile (Q1), the highest quartile of NPAR (NPAR ≥ 17.0; aHR = 1.81, 95%CI: 1.35–2.43) and NLR (NLR ≥ 3.3; aHR = 1.59, 95%CI: 1.18–2.15) were both independently associated with greater all-cause mortality risk after adjustment. In contrast, higher PLR in quartiles was not significantly associated with increased risk for mortality (Table 2).

**Table 2:** Associations between NPAR, NLR, PLR, and all-cause mortality in patients with HF

	Univariate			Multivariable		
	Crude HR (95%CI)	*P* value		Adjusted HR (95%CI)	*P* value	
In continuous						
NPAR	1.12 (1.09, 1.15)	**< 0.001**		1.08 (1.05, 1.12)	**< 0.001**	
NLR	1.09 (1.07, 1.12)	**< 0.001**		1.05 (1.02, 1.08)	**0.004**	
PLR	1 (1.00, 1.00)	**< 0.001**		1 (1.00, 1.00)	0.3	
In category						
NPAR						
Q1	Reference			Reference		
Q2	1.52 (1.16, 1.97)	0.002		1.11 (0.81, 1.52)	0.5	
Q3	1.62 (1.24, 2.10)	**< 0.001**		1.09 (0.80, 1.48)	0.6	
Q4	2.83 (2.21, 3.64)	**< 0.001**		1.81 (1.35, 2.43)	**< 0.001**	
NLR						
Q1	Reference			Reference		
Q2	1.51 (1.15, 1.98)	0.003		1.11 (0.81, 1.54)	0.5	
Q3	2.14 (1.64, 2.78)	**< 0.001**		1.26 (0.93, 1.72)	0.14	
Q4	2.91 (2.26, 3.76)	**< 0.001**		1.59 (1.18, 2.15)	**0.002**	
PLR						
Q1	Reference			Reference		
Q2	1.08 (0.84, 1.39)	0.556		1.05 (0.79, 1.40)	0.7	
Q3	1.25 (0.97, 1.60)	0.081		1.21 (0.91, 1.61)	0.2	
Q4	1.45 (1.14, 1.83)	**0.002**		1.09 (0.83, 1.44)	0.5	

Multivariable analyses were adjusted for age (in years), gender, race, BMI (category), DM, CKD, COPD, history of MI, trouble sleeping, and diuretics usage. Bold P values indicate significance

Abbreviations: NPAR, neutrophil-percentage-to-albumin ratio; PLR, platelet-to-lymphocyte ratio; NLR, neutrophil-to-lymphocyte ratio; Q, quartile; HR, hazard ratio; CI, confidence interval

### ROC analysis of NPAR, NLR, and PLR in predicting all-cause mortality in individuals with HF

**Fig. 2:**
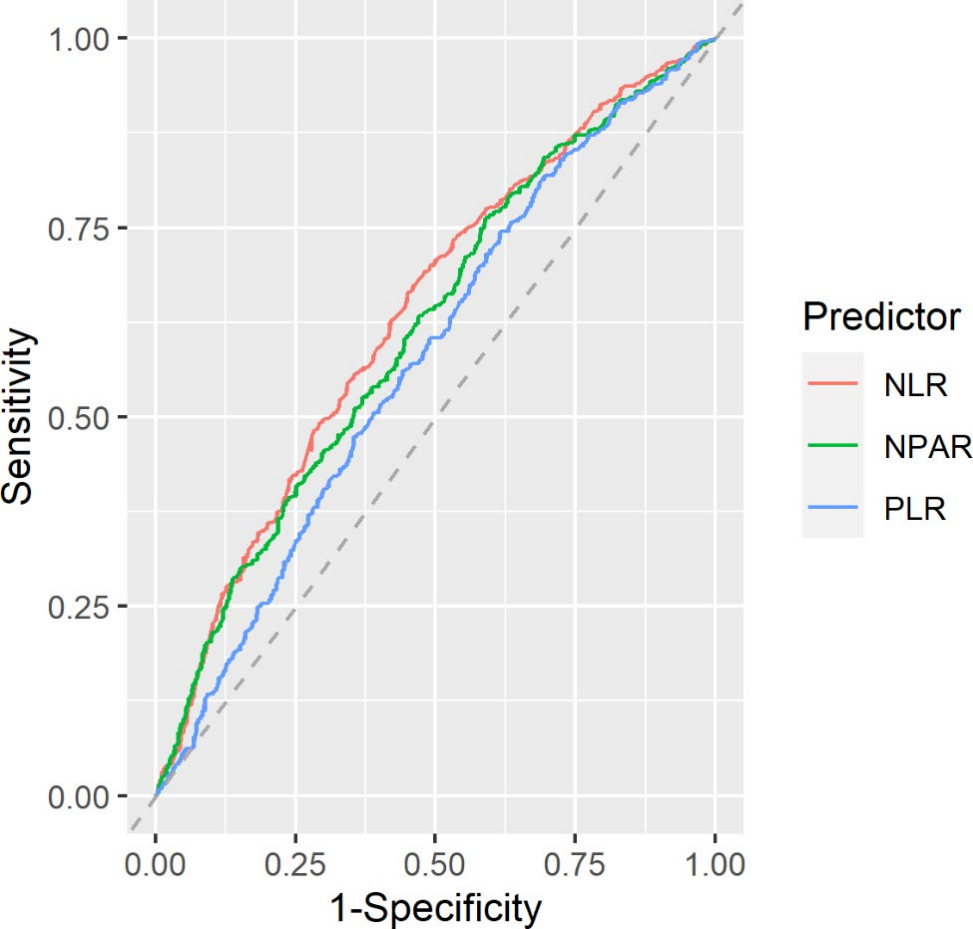
Receiver-operating characteristics (ROC) curves of NPAR, PLR, and NLR for predicting all-cause mortality

The predictive performance of NPAR, NLR, and PLR on all-cause mortality is demonstrated in Fig. 2 and Table 3. The AUC of NPAR, NLR, and PLR were 0.61 (95%CI: 0.58–0.65), 0.64 (95%CI: 0.6–0.67), and 0.58 (95%CI: 0.55–0.61), respectively (Fig. 2 and Table 3).

**Table 3:** AUC of NPAR, PLR, and NLR in predicting all-cause mortality in patients with HF

	AUC (95%CI)	Sensitivity	Specificity
NPAR	0.61 (0.58, 0.65)	34.8%	80.4%
NLR	0.64 (0.60, 0.67)	26.8%	85.3%
PLR	0.58 (0.55, 0.61)	18.7%	89.8%

Abbreviations: AUC, the area under the ROC curve; CI, confidence interval; NPAR, neutrophil percentage-to-albumin ratio; PLR, platelet-to-lymphocyte ratio; NLR, neutrophil-to-lymphocyte ratio

### Associations between NPAR, NLR, PLR and all-cause mortality among subgroups

Associations between NPAR, NLR, PLR and all-cause mortality by obese and DM status were documented in Table 4. After adjustment, per unit increase of NPAR, NLR, and PLR were significantly associated with greater all-cause mortality risk in obese and DM subgroup but not in non-obese or non-DM subgroup. The AUC of NPAR in predicting all-cause mortality was 0.65 (95%CI: 0.61–0.69) in obese subgroup and 0.63 (95%CI: 0.58–0.68) in DM subgroup (Table 4).

**Table 4:** Associations between NPAR, NLR, PLR, and all-cause mortality in patients with HF by obese and DM status

	Univariate				Multivariable	
Subgroup	AUC (95%CI)	Crude HR (95%CI)	*P* value		Adjusted HR (95%CI)	*P* value
Obese (BMI ≥ 30)						
NPAR	0.65 (0.61,0.69)	1.17 (1.12, 1.21)	**< 0.001**		1.14 (1.08, 1.19)	**< 0.001**
NLR	0.66 (0.62,0.7)	1.29 (1.22, 1.36)	**< 0.001**		1.31 (1.20, 1.43)	**< 0.001**
PLR	0.61 (0.57,0.66)	1.00 (1.00, 1.01)	**< 0.001**		1.00 (1.00, 1.01)	**< 0.001**
Non-obese (BMI < 30)						
NPAR	0.59 (0.55,0.64)	1.1 (1.06, 1.14)	**< 0.001**		1.04 (1.00, 1.09)	0.062
NLR	0.61 (0.56,0.66)	1.06 (1.03, 1.09)	**< 0.001**		1.01 (0.97, 1.06)	0.6
PLR	0.52 (0.48,0.57)	1.00 (1.00, 1.00)	0.205		1.00 (1.00, 1.00)	0.3
DM						
NPAR	0.63 (0.58,0.68)	1.14 (1.10, 1.19)	**< 0.001**		1.15 (1.09, 1.20)	**< 0.001**
NLR	0.65 (0.6,0.7)	1.22 (1.15, 1.28)	**< 0.001**		1.28 (1.18, 1.38)	**< 0.001**
PLR	0.60 (0.55,0.65)	1.00 (1.00, 1.00)	**< 0.001**		1.00 (1.00, 1.01)	**0.003**
Non-DM						
NPAR	0.60 (0.56,0.65)	1.1 (1.06, 1.14)	**< 0.001**		1.04 (0.99, 1.08)	0.093
NLR	0.62 (0.58,0.67)	1.07 (1.04, 1.11)	**< 0.001**		1.01 (0.96, 1.06)	0.7
PLR	0.56 (0.52,0.6)	1.00 (1.00, 1.00)	**0.025**		1.00 (1.00, 1.00)	0.6

Multivariable analyses were adjusted for age (in years), gender, race, BMI (category), DM, CKD, COPD, history of MI, sleep disorder, and diuretics usage. Bold P values indicate significance

Abbreviations: HR, hazard ratio; AUC, the area under the ROC curve; CI, confidence interval; NPAR, neutrophil percentage-to-albumin ratio; PLR, platelet-to-lymphocyte ratio; NLR, neutrophil-to-lymphocyte ratio; BMI, body mass index; DM, diabetes mellitus

## Discussion

Searching for the prognostic markers is vital because of the poor prognosis associated with HF. This study investigated the potential associations between biomarkers derived from the routine blood test, namely NPAR, NLR, PLR, and all-cause mortality risk in community-dwelling individuals with HF. To the best of our knowledge, this is the first study to assess the link between the novel biomarker NPAR and the long-term mortality of HF among patients living in the community. The results indicated that, after adjusting for relevant confounders, individuals with an elevated NPAR ≥ 17 (vs. < 13.2) had a 81% increased all-cause mortality risk, and those with an elevated NLR ≥ 3.3 (vs. < 1.7) had a 59% increased mortality risk during a median follow-up of 66 months. Higher PLR was not associated with a greater risk of death. Further, the predictive performance of NPAR and NLR was 0.61 and 0.64 in the entire cohort, 0.65 and 0.66 among obese individuals, and 0.63 and 0.65 among subjects with DM, all of which are regarded as poor [22], indicating that NPAR or NLR alone is insufficient to serve as long-term prognostic markers in community-dwelling patients with HF.

Inflammation plays a pivotal role in HF’s development and disease progression [23, 24]. Low-grade inflammation was recognized as a common feature of heart failure with preserved ejection fraction (HFpEF) pathology [25]. Specifically, as a result of an inflammatory stimulus, leukocytes release various inflammatory cytokines, including TNF-, IL-6, and CRP, as well as some proteolytic enzymes. These pro-inflammatory cytokines damage the myocardium, decreasing left ventricle (LV) function and subsequent HF [26].

NLR and PLR were reported to work as reproducible biomarkers in systemic inflammatory activities. NLR was continuously linked to mortality in the general population [27, 28]. For example, Song et al. demonstrate that NLR predicts mortality in the US general population. While combined with other information in different scenarios, it may be helpful for clinical risk stratification [27]. Gu et al. included 2,827 adult subjects in the NHANES database. They reported that NLR is an independent factor related to mortality in the general population. NLR’s AUC for predicting all-cause and cardiovascular mortality were 0.632 and 0.653, respectively [28].

Concerning HF, specifically, a list of studies linked NLR and PLR with the prognosis of acute HF. A retrospective study by Angkananard et al. concluded that elevated NLR on admission in patients with acute HF was independently associated with worse cardiovascular events, rehospitalization for HF, and in-hospital death in 321 patients with acute HF [29]. A recent Korean study by Cho et al. intended to investigate the application of NLR in predicting prognosis in acute HF. That study queried data from 5,625 patients in the Korean Acute Heart Failure registry and found patients in the highest NLR quartile (> 7.0) had the highest in-hospital and post-discharge three-year mortality [30]. In line with these reports, our results also showed high NLR was independently associated with increased mortality risk. However, the above-cited reports mainly focused on the hospital settings instead of individuals who lived in the community.

The prognostic role of PLR on HF outcomes is relatively limited compared to NLR, and the findings seemed inconsistent in the literature. Ye et al. reported that a higher PLR was associated with poor clinical outcomes in patients with acute HF and might be a novel marker in acute HF management [11]. Another recent study by Turcato et al. reported that PLR was independently associated with a 3-fold 30-day mortality risk after emergency department admission for acute decompensated HF [31]. On the contrary, Heidarpour et al. concluded that PLR could not be used as an independent prognostic factor among patients with acute decompensated HF [32]. Pourafkari et al. documented that PLR failed to independently predict the prognosis of acute HF [33]. Similar to some of the above-cited studies, our results suggested that PLR was not an independent predictor for long-term mortality of HF after adjusting for relevant demographic factors and subjects’ medical history.

NPAR, also derived from the routine blood test, is a relatively new marker. It is known that low serum albumin levels are an indicator of the severity of inflammation and susceptibility to infection complications [34]. A previous study reported that NPAR outperforms NLR and albumin alone in predicting stroke-associated infection [35]. Up to now, there is only one study in the literature, conducted by Hu et al., associating NPAR with the prognosis of HF. The authors evaluated 30-day, in-hospital, 90-day, and 365-day mortality in patients with HF admitted to the intensive care units (ICUs). The HR (95% CI) of the upper tertile (> 27.64) was associated with 2.29-fold 30-day mortality compared with the reference value (< 22.56), and an NPAR > 27.64 was also independently associated with 90-day and 365-day all-cause mortality risk [15], which is in line with our findings.

Although NPAR and NLR were both linked to mortality risk in the present analysis. According to the ROC analysis, the predictive performance of NPAR and NLR for all-cause mortality appears not good. Hu et al. did not assess the predictive performance of NPAR alone. In contrast, they reported AUCs for combing the Simplified Acute Physiology Score II (SAPS II) and NPAR (AUC = 0.731). They concluded that incorporating NPAR can improve the predictive ability of SAPS II ICU scores [15]. Our results reveal that a single test of NPAR alone may not be sufficient to predict long-term mortality in HF patients. However, whether NPAR could assist in the predictive ability in combining other symptom scores or traditional biomarkers still deserves further investigation.

This study has several limitations. First, the distinction of HF phenotypes and clinically significant data such as etiology of HF, symptom severity, functional categorization, ejection fractions, NT-proBNP, New York Heart Association (NYHA) class, hospitalizations, etc., were not available in the NHANES data set. These are critical factors that affect the prognosis of HF, and lacking this information limits the interpretation of the findings. Although we included years since HF diagnosis and HF medications, these variables were defined through questionnaires, and recall bias might exist. As a result, the findings must be validated in the current standard HF cohort, with such variables being further controlled for. NPAR, NLR, and PLR were not included as time-dependent variables. As they could change dynamically over time across the follow-up period, using only one measure could introduce bias. Accordingly, these limitations should be taken into account when interpreting our results.

Nevertheless, there were also several important strengths. First, the present analysis used the data from NHANES, which are drawn from a large and diverse sample of participants from the US population. The studied participants were representative of HF patients in the community. The findings are also regarded as generalizable to the overall US population. Logistic regressions and ROC analyses were conducted to evaluate the prognostic roles of the biomarkers of interest, carefully adjusting for multiple demographic and lifestyle variables that were rarely considered in hospital settings.

## Conclusions

In conclusion, our results suggest an independent association between elevated NPAR, NLR, and all-cause mortality risk in community-dwelling individuals with HF in the US. However, the predictive performance of NPAR and NLR alone on long-term mortality appears not good, indicating they are insufficient to serve as long-term prognostic markers when used alone. A future prospective study with the severity of HF being controlled for and to query the prognostic value of NPAR and NLR combined with other clinical variables are highly recommended.

## Data Availability

All related data files are publicly available from the NHANES website: https://www.cdc.gov/nchs/nhanes/index.htm.
